# An investigation of the reproducibility and usefulness of automatic couch motion in complex radiation therapy techniques

**DOI:** 10.1120/jacmp.v3i1.2591

**Published:** 2002-01-01

**Authors:** Milton K. Woo, Bryan Kim

**Affiliations:** ^1^ Department of Medical Physics Toronto‐Sunnybrook Regional Cancer Centre Ontario Canada M4N 3M5; ^2^ Department of Medical Biophysics Department of Radiation Oncology University of Toronto Ontario Canada M4N3M5

**Keywords:** treatment couch, automated treatment techniques, quality assurance

## Abstract

Radiation therapy techniques that incorporate multiple couch motions are becoming more common, and they often involve an increasing level of complexity along with a need for automatic motion. The reproducibility of automatic couch motion is thus a growing concern. In this work we carried out various tests to assess the automatic motion of a commercial treatment couch, including tests to evaluate the digital readout reproducibility, as well as an independent verification of the reproducibility of the couch positions on repeated motions, using phantoms as well as a volunteer subject. It was shown that the couch motion is highly reproducible, with no discomfort to the patient, and can greatly improve treatment times as well as reduce errors in couch positioning.

PACS number(s): 87.52.–g, 87.53.–j

## INTRODUCTION

With the advent of precision conformal radiation therapy, treatment techniques are becoming more complex. An example is the intensity modulated radiation therapy (IMRT) technique, which could involve tens or even hundreds of field segments, as well as multiple gantry angles. Furthermore, techniques involving couch motion are slowly emerging. Examples are IMRT treatments involving noncoplanar beams, and/or multiple isocentres, as well as specialized applications such as the HD270 feature on the Siemens linear accelerators, which utilizes couch movement to create a virtual micro‐multi‐leaf‐collimator field.

Treatment techniques involving couch motion are certainly not new. The slice‐based IMRT^1^ ^,^ ^2^ technique has been in use for years. The dynamic mode of radiosurgery has also been in standard practice for many years.^3^ These specialized techniques normally use complicated custom‐designed equipment to control the couch as well as to immobilize the patient.

Although many modern treatment couches are capable of fully automated motions, the use of automatic couch motions for routine treatment is still not prevalent. One main reason is the concern whether the couch can reproducibly be driven to the programmed position. Another is the safety aspect of moving the patient remotely, without a radiation therapist being present in the treatment room. It is then the purpose of this particular study to address these quality assurance issues, with the objective of justifying and promoting the increased use of automated motion of the treatment couch.

Some works exist in the literature concerning the accuracy and reproducibility of treatment couch motions. One paper assesses these aspects of the Siemens ZXT couch in its prototype design stage.^4^ That work is concerned with the application of automatic couch motions from the point of on‐line correction based on electronic portal imaging verification, and involves relatively small adjustment of the couch, on the order of less than 2 cm. Our particular investigation attempts to be more general, and looks at small couch movements for applications such as image‐guided radiation therapy (IGRT) adjustments or the HD270 application mentioned above, as well as larger couch movements such as multiple isocentre or noncoplanar techniques in IMRT, and also the more conventional techniques utilizing multiple isocenters, such as different source‐to‐surface distances (SSD) fields, or certain breast techniques. In addition, much work is done in the area of patient positioning accuracy and its impact,^5^ but this is not the subject of our study here, nor is the process of verifying the couch specifications, which is a standard part of commissioning of the couch. Instead, we emphasize the reproducibility of the automatic couch motion, in order to promote the practice of controlling the couch remotely, as opposed to the traditional manual control practice.

## METHODS

The treatment couch used in this investigation is the Siemens ZXT couch with version 7 software, as part of the package of a Primus linac. The ZXT couch is a scissors‐jack‐typed couch and can be remotely controlled in all rectilinear *x, y,* and *z* directions, as well as rotation about the mechanical isocenter. The digital readout has a resolution of 0.1 cm in all rectilinear motions and 0.1 degree in isocentric rotation.

In the automatic control mode, linac and couch motions are specified in the record‐and‐verify software module, Primeview, and downloaded into the linac control console. Primeview also offers a “sequence” mode, whereby fields are downloaded and executed sequentially and automatically without the need for intervention. With the gantry and couch motions, the first motion in the sequence has to be enabled manually, but afterwards the motion will be automatic. The motion could be stopped by hitting the manual stop buttons or any key on the keyboard.

The couch coordinates in a sequence are relative, in order to allow the patient position to be set up to a tattoo, but all subsequent motions will be relative to the first position as prescribed in the plan.

The following tests were carried out with the main objective to assess the automatic and remote‐controlled aspects of the couch, as compared to the standard manual‐controlled process. First, the reproducibility of couch positions as indicated by the couch digital readouts was tested. This assessed how reproducibly the couch could be positioned correctly to a set of preprogrammed coordinates. This automatic procedure emulated the manual process where a therapist would position the couch using the same digital readouts. In the second test, the reproducibility of the positioning of markers on a phantom under automatic couch movement was studied using room lasers as the reference. This was a more stringent test than the first one, but could be carried out only for a smaller set of couch positions for which the room lasers fell on the surface of the phantom. The third test replaced the phantom with a human subject volunteer to simulate a closer clinical situation. The other tests on patient comfort and treatment time assessed the impact on these factors when the automatic couch movement replaced the standard manual procedure. The following describes the individual tests in greater detail.

### A. Digital readout test

This test was done by executing a sequence of five couch motions with positions relatively far apart to cover a wide range of couch movements. A sequence consisting of five couch positions, repeated ten times, was set up in Primeview. This whole sequence of 50 segments incorporating couch motions was downloaded sequentially to the Primus console and executed individually. At each segment the digital readout on the console as well as at the couch was noted.

### B. Couch position reproducibility

For this second test, the Rando phantom was placed on the couch and five markers placed on it such that a marker was aligned with the right horizontal room laser crosshair at each of the five couch positions. These five couch positions were sequenced through nine times to assess the reproducibility of the couch control function. The Rando phantom was positioned for the treatment of two typical sites, namely head‐and‐neck and prostate, where the couch table top was extended further out, in order to assess the effect of any sagging of the table top.

### C. Patient positioning on couch

For this test, the measurements done above using the Rando phantom were repeated using a volunteer subject. In addition, the volunteer was asked to note any “jarring” motion during the couch movements. Currently, the segment sequence cannot be programmed to run completely through with no dose delivered, so a semiautomatic sequence was simulated by loading each segment individually.

In conjunction with the study of any jarring effect, the motion of the couch was observed closely for a few segments during the entire motion, in particular, noting any jarring motion at the beginning and the end of the motion.

### D. Remote monitoring

Since, as mentioned above, the autosequence cannot be run without having a beam on, a video camera was attached to the treatment couch and the measurements carried out with the Rando phantom were repeated and monitored for a complete sequence of fields. The camera would also be useful to verify positioning for actual patient treatment.

### E. Timing consideration

In addition to positioning reproducibility, the amount of time taken for the couch motions was also noted in order to compare to the process of manually adjusting the couch by going in and out of the treatment room.

## RESULTS

### A. Digital readout test

Table I shows the programmed coordinates of the five couch positions used for this test. The digital readout data for each of the ten repeated sequence of the five positions all fall within ±0.1cm or ±0.1° of the programmed values of the rectilinear or isocentric motions, respectively, and the standard deviation of each of the five positions was evaluated to be less than the resolution of the readouts of 0.1 cm and 0.1°, respectively.

**Table I:** Results of digital readout tests. Standard deviation for rectilinear motion <0.1cm. Standard deviation for isocentric motion <0.1°.

	Lateral	Longit	Vertical	Isocentric
Pos 1	10.0 cm	10.0 cm	10.0 cm	0.0°
2	4.0 cm	11.0 cm	15.0 cm	10.0°
3	12.0 cm	9.0 cm	3.0 cm	330.0°
4	1.0 cm	14.0 cm	17.0 cm	40.0°
5	16.0 cm	2.0 cm	8.0 cm	355.0°

### B. Couch position reproducibility

For this set of measurements, the laser crosshair was aligned at each of the five markers initially, and then the couch was repositioned for the nine subsequent moves. For each position, the crosshair was observed to either fall on the markers or at the edge of the markers. Since the laser crosshair was approximately 0.1 cm in width the reproducibility was again determined to be less than 0.1 cm.

The data for both the head and neck position and the prostate position, where the couch was extended further, showed no discernible differences.

### C. Patient positioning on couch

When the Rando Phantom was replaced by a volunteer subject, it was found that the process of placing the markers for each couch position to line up with the laser crosshair would introduce errors on the markers that have already been placed on the subject, due to the unavoidable motion during marker placement. Hence, the couch coordinates were slightly adjusted to align with all the markers after all markers have been placed, and these new coordinates were used as the reference. In addition, only three couch positions were used, and the sequence repeated three times, in order to reduce the time that the subject had to stay still. With these adjustments, the reproducibility of the marker positions was again found to be less than 0.1 cm. One observation by the volunteer was that when the couch was moved manually to adjust the laser alignment, the jarring motion was quite noticeable. In contrast, during remotely controlled automatic couch motion, the motion was relatively smooth, with a slower speed at the beginning and end of the motion. It appears clear that any added uncertainty would be due to the inadequate immobilization of the patient, rather than any extra jarring motion from the automatic couch movement.

### D. Remote monitoring

With the remote camera set up to monitor the laser crosshair on the Rando Phantom, the automatic sequence used above was cycled through completely for ten times without any error (i.e., the digital readouts were positioned within the tolerance of ±0.1cm or ±0.1°). In addition the laser crosshair was observed to fall within the 0.1‐cm margin, as found similarly above.

### E. Timing consideration

For the sequence of five couch positions with ten monitor units delivered for each beam, the automatic sequence took 5 min. In contrast, the manual process of positioning the couch for each segment took 8 min.

## DISCUSSION

Automatic couch motion has always been a concern in radiation therapy techniques, both for the safety of the patient and the reproducibility of the motion. Many safeguards, such as touch‐sensitive interlocks, have been built into the modern design of linac couches and gantry structures. However, having the patient moved remotely has always introduced some concern, which might be alleviated with experience and exhaustive quality assurance testing. Further reassurance is gathered by referring to the literature, such as the dynamic radiosurgery technique in Ref. 3, which has been carried out for many years, where both the gantry and couch would move simultaneously and continuously while the radiation beam is on. The successful delivery of the technique on hundreds of patients should alleviate some of the safety concern for remote motion of the couch.

One difference of the above technique is the extensive use of immobilization devices, such as a head frame. The results of this present work indicate that automatic motion of the couch would not contribute any extra uncertainty to patient positioning. This is logical since currently the patient would be moved by the therapist via manual control of the couch inside the treatment room, but the set‐up tattoos are generally not rechecked at the new set‐up position, so no error correction is involved even for the manual process. In this work we showed that the couch motion has a minimal jarring effect, with gradual adjustment of couch speed during motion. In fact, the reproducibility could actually be increased since the setup is done automatically using input planning data, instead of manual couch positioning, so that human error is minimized.

It should be noted that many conventional techniques, such as techniques using different SSD's, or some complicated breast techniques that use multiple isocenters or certain noncoplanar beam techniques, already involve manual couch motions. These techniques could benefit from this study on the reproducibility of automatic couch motions by automating the manual process.

With increasingly complex techniques, automatic linac motion is becoming not only desirable, but almost a necessity. This paper addresses the aspect of the couch motion in terms of reproducibility, but does not address the issue that the couch coordinates are programmed correctly. This latter issue may need to be addressed to further assure that the linac control system is carrying out the motions as intended. This is especially important when the treatment setup has to be downloaded from the treatment planning system and transferred over several computers, such as the record and verify computer system, to the actual linac control system. The integrity of this beam data is of great concern, and an independent real‐time monitoring system is a good quality assurance tool in this aspect. As part of our program to develop a comprehensive IMRT and advanced automatic high‐precision radiation therapy program, we are also developing a real‐time linac and couch position monitoring system, as well as a beam shape verification system, both using the Electronic Portal Imaging System to detect the actual delivered beam image and correlate it to the planned beam shape and linac and couch positions. These efforts will be reported in separate papers.

## CONCLUSION

In this work we carried out various tests to assess the automatic motion of a commercial treatment couch, including tests to evaluate the digital readout reproducibility and also an independent verification of the reproducibility of the couch positions on repeated motions, using the Rando phantom as well as a volunteer subject. It was shown that the couch motion is highly reproducible, with no discomfort to the patient, and can greatly improve treatment times as well as reduce errors in couch positioning. This work then establishes the practicality of using automatic couch motion to reduce treatment time, for both conventional and advanced complex techniques such as IMRT, and forms the basis of an advanced real‐time quality assurance system for monitoring image guided radiation therapy.
